# Correlative light and scanning electron microscopy (CLSEM) for analysis of bacterial infection of polarized epithelial cells

**DOI:** 10.1038/s41598-019-53085-6

**Published:** 2019-11-19

**Authors:** Carina Kommnick, Andrea Lepper, Michael Hensel

**Affiliations:** 1 Abt. Mikrobiologie, Universität Osnabrück, Osnabrück, Germany; 2 CellNanOs – Center for Cellular Nanoanalytics Osnabrück, Fachbereich Biologie/Chemie, Universität Osnabrück, Osnabrück, Germany

**Keywords:** Optical imaging, Cellular imaging, Pathogens

## Abstract

Infection of mammalian host cells by bacterial pathogens is a highly dynamic process and microscopy is instrumental to reveal the cellular and molecular details of host-pathogen interactions. Correlative light and electron microscopy (CLEM) combines the advantages of three-dimensional live cell imaging with ultrastructural analysis. The analyses of adhesion to, and invasion of polarized epithelial cells by pathogens often deploys scanning electron microscopy (SEM), since surface structures of the apical brush border can be analyzed in detail. Most available CLEM approaches focus on relocalization of separated single cells in different imaging modalities, but are not readily applicable to polarized epithelial cell monolayers, since orientation marks on substrate are overgrown during differentiation. To address this problem, we developed a simple and convenient workflow for correlative light and scanning electron microscopy (CLSEM), using gold mesh grids as carrier for growth of epithelial cell monolayers, and for imaging infection. The approach allows fast live cell imaging of bacterial infection of polarized cells with subsequent analyses by SEM. As examples for CLSEM applications, we investigated trigger invasion by *Salmonella enterica*, zipper invasion by *Listeria monocytogenes*, and the enterocyte attachment and effacement phenotype of enteropathogenic *Escherichia coli*. Our study demonstrates the versatile use of gold mesh grids for CLSEM of the interaction of bacterial pathogens with the apical side of polarized epithelial cells.

## Introduction

Microbial pathogens have evolved sophisticated mechanisms to manipulate cellular functions of their mammalian hosts. For example, gastrointestinal bacterial pathogens adhere to, and occasionally invade cells of the intestinal epithelium during infection. Bacterial adhesion to, and invasion of the intestinal epithelium are fast and highly dynamic events. Visualization of the host-pathogen interplay in the intestinal environment remains the ultimate goal, but is restricted by the limited access to these organs and spatiotemporal challenges. Thus, infection models in cell or tissue cultures represent an important experimental tool to study gastrointestinal pathogenesis on a cellular and molecular level. *In vitro* organ cultures (IVOC), intestinal organoids, or polarized epithelial cell cultures provide excellent experimental accessibility, for example for live cell imaging or ultrastructural analyses. Polarized epithelial cell lines are attractive models for reassembly of epithelial layers. Canine kidney MDCK cells and human colonic Caco-2 cells form monolayers with cell-cell contacts, polarization of apical and basolateral sides, and maintain functional barriers. Especially cells of clone Caco-2 BBe1 form brush borders comparable to that of the intestinal mucosa^1^.

For investigation of bacterial interactions with epithelial layers, correlative light and electron microscopy (CLEM) is the method of choice. CLEM combines the advantages of high-dimensional live cell imaging (LCI), allowing highest temporal analysis, with the ultrastructural resolution of electron microscopy (EM). While fast LCI provides insights in dynamic cellular processes, like the rearrangement of cytoskeleton over time, ultrastructural analysis by EM can be performed at any fixed time point. Using transmission electron microscopy (TEM) or scanning electron microscopy (SEM), intracellular organelles and extracellular surfaces, respectively, may be imaged with the highest spatial resolution and within their cellular context. Especially analyses of dynamic microbial adhesion and invasion can benefit from combining LCI with SEM in CLEM approaches.

In this study, we deployed models of infection of polarized epithelial cells by three important gastrointestinal pathogens that also serve as key model organisms for bacterial pathogenesis.

*Salmonella enterica* serovar Typhimurium (STM) induces its uptake in non-phagocytic cells by rearranging the host cell actin cytoskeleton reviewed in^2^. By contact to the apical surface of polarized epithelial cells in the intestine, bacterial effector proteins are translocated into the host cell through a type III secretion system (T3SS), encoded by genes on *Salmonella* pathogenicity island 1 (SPI1)^3^. SPI1-T3SS effector proteins lead to accumulation of host cell F-actin at the contact site of STM, inducing brush border effacement and membrane ruffles that engulf the pathogen, resulting in macropinocytosis-like internalization. LCI studies revealed that this process is highly dynamic, and results in distinct morphologic changes of host cell apical surface within seconds^4^.

*Listeria monocytogenes* is a Gram-positive foodborne pathogen that invades intestinal cells by the zipper mechanism reviewed in^5^. Here, multiple interactions of the bacterial surface protein Internalin A with mammalian E-cadherin cause adhesion to and clustering of host cell receptors, ultimately inducing signal transduction events resulting in the internalization of the pathogen^6,7^.

Enteropathogenic *Escherichia coli* (EPEC) is a pathogenic variant of intestinal commensal *E. coli*. EPEC deploys distinct adhesion factors for the loose and intimate adhesion to the apical side of epithelial cells reviewed in^8^. Intimate adhesion is mediated by the T3SS effector protein Tir (Translocated Intimin Receptor) and its cognate bacterial ligand Intimin. Tir-Intimin interaction allows tight adhesion to the apical side of enterocytes, leads to effacement of the brush border, and induces formation of pedestals, F-actin based membrane protrusions formed below adhering EPEC^9,10^.

The correlative microscopy of pathogen interactions with polarized epithelial cells imposes specific experimental challenges, since conventional CLEM approaches are not applicable. Previous CLEM methods established workflows mostly to re-identify regions of interest (ROI) of host-pathogen-interactions on non-polarized cells, like HeLa, which are cultured as well separated single cells^11–13^. For such CLEM approaches, coordinate systems on dish bottoms can be used to relocate specific single cells. The coordinates are visible by light microscopy and transferrable to the resin block surface by sample embedding, mostly done for TEM. This was already established to analyse the formation of *Salmonella*-induced filaments in HeLa cells^14,15^. For correlative SEM studies of separated single cells, also patterning of the dish bottom was used, like carbon coating or metal sputtering of dish bottoms by distinct pattern masks^16^, or stochastic gold micro-patterns^17^. For relocating specific areas, these patterns have to be clearly visible in SEM. However, this is problematic with confluent monolayer cells, since dish bottom patterns are completely covered by the cells.

In this report, we introduce an easy and robust method for correlative light and scanning electron microscopy (CLSEM) of monolayer cells by utilizing gold mesh grids coated with carbon and formvar (C + F) film. While polarized epithelial cells completely overgrow the C + F film in a monolayer, the gold mesh grid can still be visualized in both light microscopy and SEM. Using landmarks in the grid centre for orientation, areas of up to 200 µm² can be chosen as ROI in live cell imaging by simply counting holes of the mesh grid. After EM sample preparation, gold mesh grids can be easily transferred to a carrousel holder for further SEM analysis. As specific setups combing fluorescence imaging and electron microscopy may require expensive software, and transfer of coordinates is not available in all facilities, the EM grid provides a convenient CLSEM sample carrier. By utilizing gold mesh grids for CLSEM, we were able to unravel the morphologic fate of the apical brush border of polarized monolayer cells during bacterial invasion at distinct time points.

## Results

### Gold mesh grids with carbon + formvar film as carrier for correlative workflows

We developed workflows that allow the correlation of LCI of infections of polarized epithelial cells by bacterial pathogens with ultrastructural analyses by SEM. For this, a suitable carrier for transfer between the imaging modalities was required. We found that standard EM gold mesh grids coated with a C + F film can be used as convenient carriers for CLSEM. The grids are routinely used for TEM applications, therefore designed to withstand relative harsh procedures such as fixation by glutaraldehyde, ethanol dehydration and chemical drying. While inexpensive copper grids inhibited growth of epithelial cells, the more expansive gold grids showed excellent biocompatibility, and were used throughout this work. When grown on the gold mesh with C + F film facing upwards, epithelial monolayer cell lines MDCK and Caco-2 BBe1 showed no differences in brush border differentiation or generation times compared to cells seeded in ordinary cell culture plates (data not shown).

For attaching mesh grids to bottoms of culture dishes suitable for light microscopy, we used gelatine as adhesive. By this, grids did not detach from the bottom while pouring cell culture medium in the dish. Also, gelatine seals the borders of grids to prevent undesired growth of cells between grid and dish bottom. As the grids have to be transferred to a SEM carrousel holder later in the process, they can be easily detached from dish bottoms using forceps. After attaching mesh grids, epithelial cells were seeded on top of the grid and allowed to form monolayers. We used gold mesh grids of 200 µm^2^ square holes with landmarks in the grid centre that enable registration of a ROI during LCI for further correlation (Fig. S1). While grids with a larger mesh were also applicable, 200 µm^2^ holes filled the entire imaging area when using 63x objective in spinning disc confocal microscopy (SDCM). After chemical fixation of the cells, samples were prepared for SEM. By transferring the grids to a carrousel holder, analyses of cell surfaces were performed by SEM. Re-identification of specific mesh areas allowed correlation of cellular dynamics visualized by fluorescence microscopy with ultrastructural micrographs.

**Figure 1:**
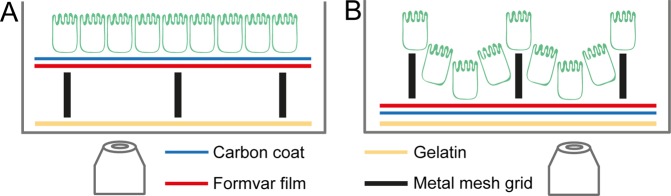
Schematic side view of carbon + formvar film orientation on mesh grid with seeded monolayer cells in dish. (**A**) A mesh grid (black bars) with C + F film (C, blue line; F, red line) facing upwards provides an even surface for monolayer cells (green symbols) to grow and differentiate homogeneously, but the gap with size of metal mesh between cells and objective (eye, grey) complicates focusing during fluorescence microscopy. As the mesh grid is covered by the monolayer after dehydration and drying for SEM, a STEM detector is required to re-identify areas of interest. (**B**) A mesh grid with C + F film facing downwards provides minimal gap between cellular monolayer and objective, leading to better and more stable focusing during imaging. Due to its distinct topographic differences at the edges of each mesh hole, growth of monolayer cells is uneven. In SEM, re-identification of specific holes does not require specific detectors, as the metal mesh is clearly visible by standard detectors.

If mesh grids are used as CLSEM carrier, defining the orientation of the C + F film on mesh grids is crucial. Before attaching the grid onto a dish bottom, one has to decide if an evenly distributed monolayer is essential for further analysis, as two grid orientations are possible (Fig. 1). When cells are seeded on grids with the C + F film facing upwards, there is a larger gap between the monolayer and the dish bottom, which has as minimum the height of the metal mesh (6–25 µm) (Fig. 1A). This space may allow vertical movement of the film, resulting in defocusing during fluorescence imaging and loss of positions. Growth and differentiation of cellular monolayers is more even without topographic disruptions by the mesh, and provides comparable cell conditions independent of the mesh area. However, since the mesh grid is completely covered by the cell monolayer, in SEM it cannot be identified without using a STEM detector.

When cells are seeded on grids with the C + F film facing downwards, there is only a small gap between the monolayer and dish bottom (Fig. 1B). This allows more stable focusing during fluorescence imaging. Additionally, mesh grid areas can be identified more easily by SEM. However, cells at the edges of the metal mesh have distinct topographic differences compared to cells on C + F film in the centre. This could lead to different growth behaviour of cells depending on their location. Special care should be taken to avoid areas where single cells attach between mesh grid and dish bottom after seeding. This might happen when the mesh grid is not completely attached to dish bottom by gelatine, or the C + F film is destroyed in distinct mesh areas. By growing into monolayer patches, the cells under the grid increase the distance of grid to dish bottom, thus decreasing visibility of the monolayer grown on the grid, and complicating focusing for imaging. These patches in between cannot be prevented completely, thus, the respective mesh areas have to be excluded of further analysis.

For the subsequent use in infection studies, the differentiation of epithelial cells into a highly polarized monolayer is of critical relevance. Quantification of transepithelial electrical resistance as used for cells grown on transwell filter insert could be applied to cells grown on mesh grids. To control monolayer formation and differentiation, we analysed formation of tight junctions (TJ) using immunostaining for TJ protein ZO-1. In MDCK and C2BB1 cells grown on mesh grids ZO-1 was present in a continuous belt at the contact area to neighbouring cells (Fig. S2). This continuous distribution was observed for cells in direct contact with the carbon coat or the formvar layer, thus independent from the orientation of the C + F film. Together with the high density of microvilli observed in the subsequent analyses, the formation of tight junctions indicates the efficient polarization of epithelial cells on mesh grids.

### Live cell imaging with gold mesh grids at various durations of bacterial infections

**Figure 2:**
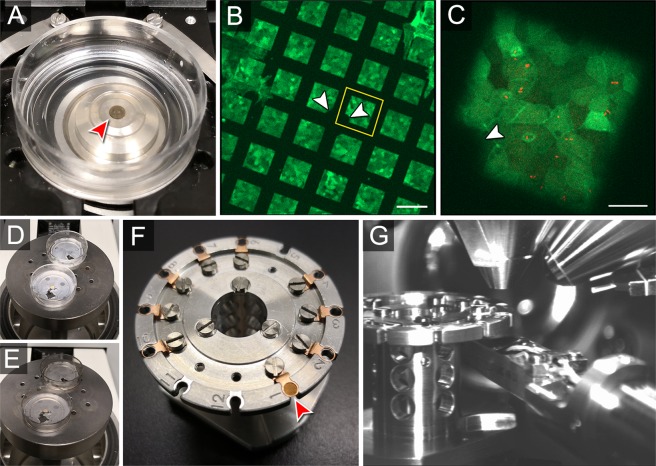
Workflow of correlative live cell imaging and scanning electron microscopy with gold mesh grids. (**A**) Live cell imaging (LCI) of STM infection of host cells on a mesh grid (red arrowhead) placed in a FluoroDish. The dish was transferred to adaptable mount on the motorized stage for spinning disc confocal microscopy (SDCM). The objective was positioned directly under the grid. Prior to defining imaging areas in the software, the lid was removed to avoid loss of defined coordinates by accidently touching. (**B**) Fluorescence image of Lifeact-eGFP MDCK cell monolayer grown on C + F film using a 10x objective. Distinct mesh holes were defined as region of interests (ROI) for LCI (yellow square). The asymmetric landmarks (white arrowheads) in the grid centre are essential for further relocalization. (**C**) Magnified ROI with 40x objective, where single cells within the monolayer were identified. A small notch in left corner of mesh area serves as visible landmark (white arrowhead). The corresponding image sequence recorded over 17 min is shown in Movie 1. (**D**) To prevent charging of biological samples during SEM imaging, grids with dehydrated monolayer cells were carbon coated by using a sputter device specified for thin carbon layer. If the sputter chamber provides enough space, grid samples are sputtered in their cell culture dishes. By this, the risk of collecting dust particles on cellular structures or losing entire grids is reduced. (**E**) After completion of carbon coating, samples appear darker. (**F**) The mesh grid was transferred to a grid carrousel holder (red arrowhead), providing several sample positions to sequentially image multiple grids. (**G**) For imaging of mesh grids with C + F film facing upwards, the STEM detector (bottom right) was used to identify orientation marks in grid centre. Therefore, the STEM detector was transferred from its parking position into the vacuum chamber, and the sample in carrousel holder was positioned manually over the detector. Scale bars, (**B**) 100 µm, (**C**) 20 µm.

Small landmarks in centre of the mesh grids are essential for the use as CLSEM carrier. While mesh holes were clearly visible by bright field or fluorescence microscopy, landmarks were used to locate holes of interest for imaging analysis (Fig. 2). Depending on the brand of mesh grid used, various forms of asymmetric landmarks are located in the central crossing of the mesh bars. By using the landmarks for orientation, mesh holes are defined by locating the X and Y positions relative to the centre. The irregular shape of cells in monolayers usually allows an easy visual orientation within a mesh hole, as shown below for polarized monolayers of MDCK and Caco-2 BBe1 cells. There is no need for special setups, as simple counting of mesh holes is basis for further correlations. The coordinates of mesh holes selected for LCI were transferred to a grid template with predefined orientation marks. For a template for recording coordinates in 200 mesh grids, see Fig. S1. For mesh holes in the vicinity of landmarks, an overview image at lower magnification was sufficient for relocation. By this, the coordinates are easily accessible on all devices.

To test the stability of monolayer cells grown on gold mesh grids, we performed imaging for 6 h with time intervals of 15 min between recording image stacks for the fluorescence channels with laser excitation at 488 nm and 561 nm by SDCM. Repeated rounds of imaging did not affect integrity of epithelial monolayers. We did not observe increased frequency of apoptotic cells, or detachment of parts of monolayers grown on the formvar film or gold mesh. The mechanic stability during sample processing was comparable for cell monolayers grown on mesh grids and for cells grown of other supports such as cover slips.

### Relocation of mesh holes by scanning electron microscopy

Most SEM are equipped with InLens SE and SESI detectors, whereas a STEM detector is a specific addition to the system (Fig. 2). Mesh holes of gold grids for correlative orientation can be visualized by the standard detectors InLens SE and SESI, and STEM detector, depending on the characteristics of monolayer cells, and especially the orientation of C + F film on the grid.

**Figure 3:**
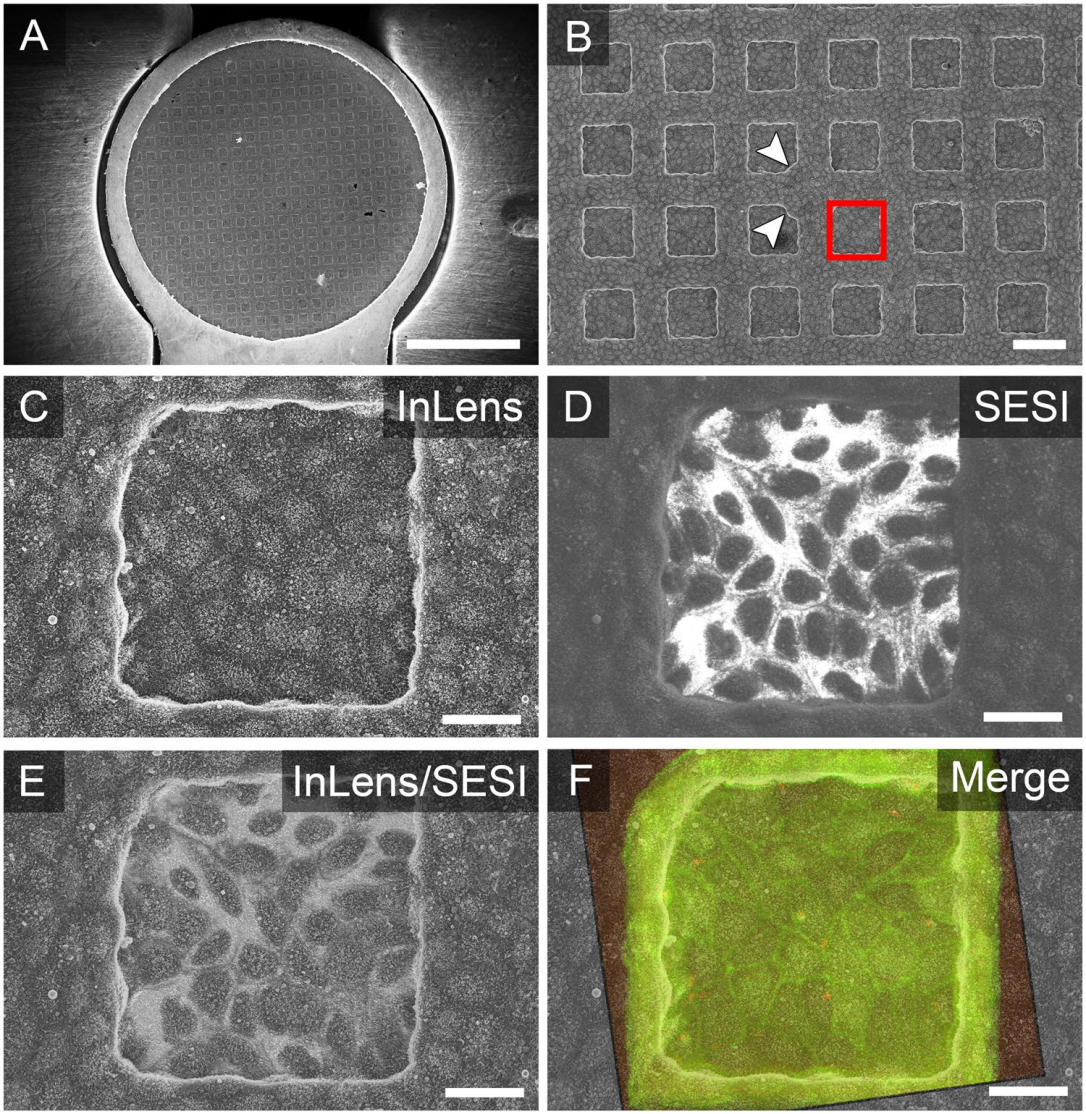
Correlation of Lifeact-eGFP MDCK cell monolayers grown on mesh grid with carbon + formvar film facing downwards. (**A**) A mesh grid was inserted into a SEM carrousel holder with already identifiable areas at low magnification. (**B**) Monolayer cells have grown on C + F film areas with a metal mesh on top. In grid centre, landmarks of metal mesh with small notches or cropped edges in corners are visible (white arrowheads). On an area of interest (red square), prior LCI of STM infection was performed. (**C**) Surface imaging was done by the InLens SE detector with EHT of 4 kV. Topographic differences are clearly visible at borders of holes, which should be omitted for correlative imaging. The growth behaviour of monolayer cells in the centre of holes was comparable to growth on the bottom of cell culture dishes regarding differentiation state and homogeneity. (**D**) Using the SESI detector with EHT of 6 kV, cell outlines become detectable more easily. (**E**) Overlay of images from InLens and SESI detectors. (**F**) Additional overlay with the fluorescence modality from LCI. Cell outlines in LCI modality, visible due to Lifeact-eGFP, matching cell outlines visualized by SESI detector. Scale bars, (**A**) 1 mm, (**B**) 100 µm, (**C**–**F**) 20 µm.

If monolayer cells were seeded on a mesh grid with C + F film facing downwards (Fig. 1B), standard SEM detectors were sufficient to visualize mesh holes (Fig. 3). To re-identify the centre area with landmarks, the estimated centre was focused at 10,000-fold magnification. Due to visible topographic differences at mesh edges, the orientation of mesh grid was identified by the InLens SE detector. Using the SESI detector, the growth pattern of individual cells in the monolayer was identified more easily. This was helpful for the subsequent correlation with fluorescence images. To identify the mesh holes more distinctly, the kV of electron high tension beam (EHT) was increased over 4 kV up to 10 kV for better electron signal. To prevent charging, use of relative high EHT was kept as short as possible.

**Figure 4:**
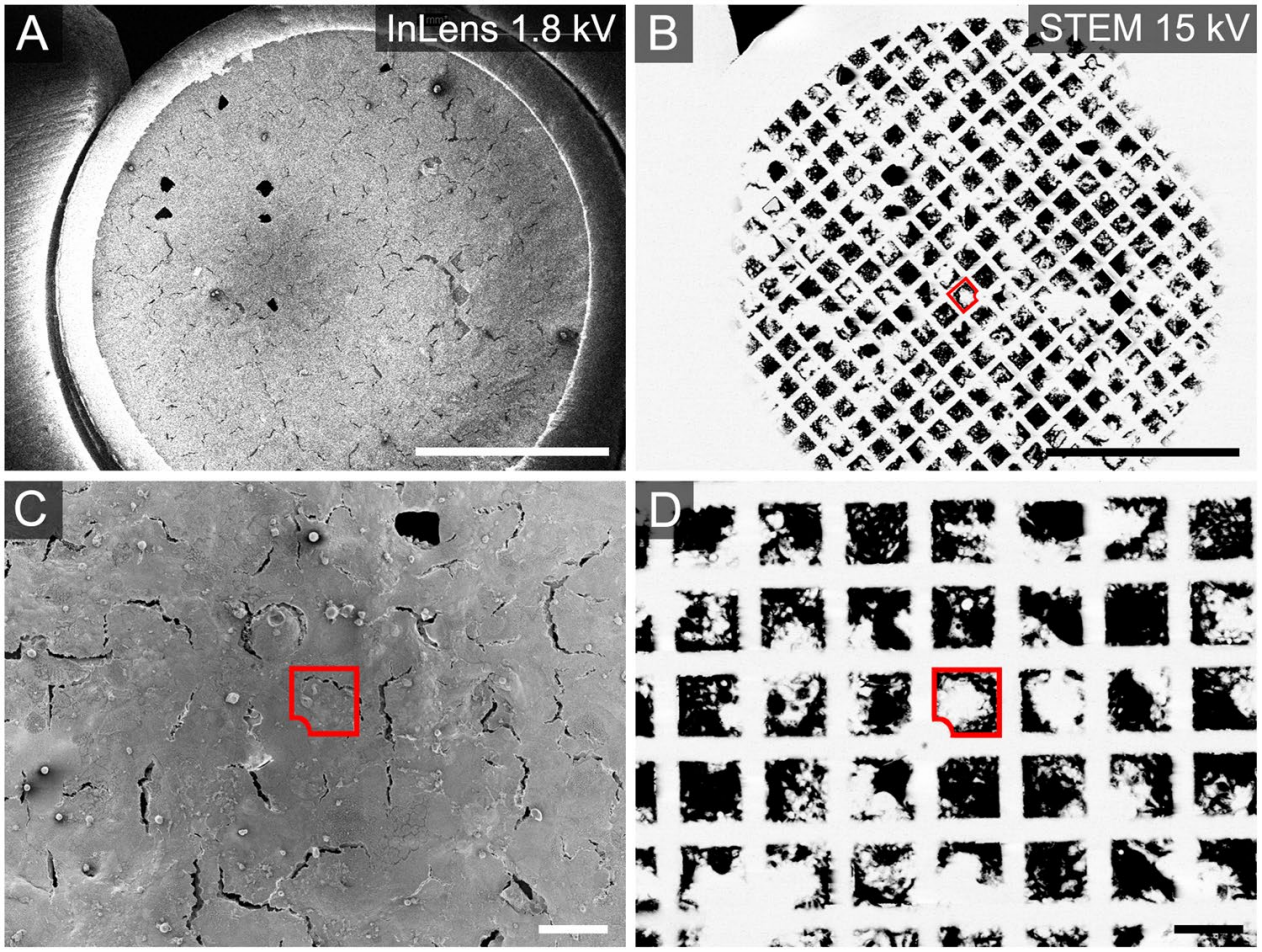
Correlation of Lifeact-eGFP Caco-2 BBe1 monolayer cells grown on mesh grid with carbon + formvar film facing upwards. (**A**) A mesh grid was inserted into the SEM carrousel holder with no identifiable areas in low magnification. Imaging was performed with low ETH 1.8 kV to avoid damage of cells. The monolayer grew homogeneously on C + F film without topographic differences. (**B**) Use of the STEM detector allowed to re-identify specific mesh holes. To visualize the metal mesh underlying the cellular monolayer, ETH was increased to 15 kV as short as possible to find landmarks in grid centre. The best recognizable mesh hole has a small notch in one corner (red square). (**C**) Area of interest was imaged by InLens SE detector, correctly identified due to landmarks by STEM detector in (**D**). Scale bars, (**A**,**B**) 1 mm, (**C**,**D**) 100 µm.

If cell monolayers developed on mesh grids with C + F film facing upwards (Fig. 1A), topographic landmarks were absent. Thus, the STEM detector was the only option to visualize underlying mesh holes (Fig. 4). The STEM detector was also selected if Z dimensions of the monolayer exceeded 13 µm, e.g. for highly columnar Caco-2 BBe1 cells, as mesh holes were not be recognizable by InLens or SESI detectors due to distinct differentiation status. By using a STEM detector with EHT of 15 kV, the gold mesh was visible due to backscattered electrons (Fig. 4B,D, white lines), while areas with lower electron density, like monolayer grown on C + F film, appeared dark (Fig. 4B,D, black squares). Again, to prevent charging, the use of relatively high EHT was kept as short as possible.

When a mesh hole with landmarks was relocated, the detector was switched to InLens SE with EHT of 1.8 kV to 4 kV. Mesh holes with ROI defined in LCI were relocalized according to grid templates and overview images.

### Correlating live cell imaging with scanning electron microscopy modalities for morphometric surface analyses

We were interested in the morphological changes of the apical side of polarized epithelial cells during infection by bacterial pathogens, and the dynamics of the host cell F-actin cytoskeleton. To follow the spatiotemporal dynamics, we generated polarized epithelial cell lines that permanently express Lifeact-eGFP. Lifeact specifically interacts with F-actin^18^, allowing the visualization of F-actin dynamics in living cells, and during bacterial infection.

**Figure 5:**
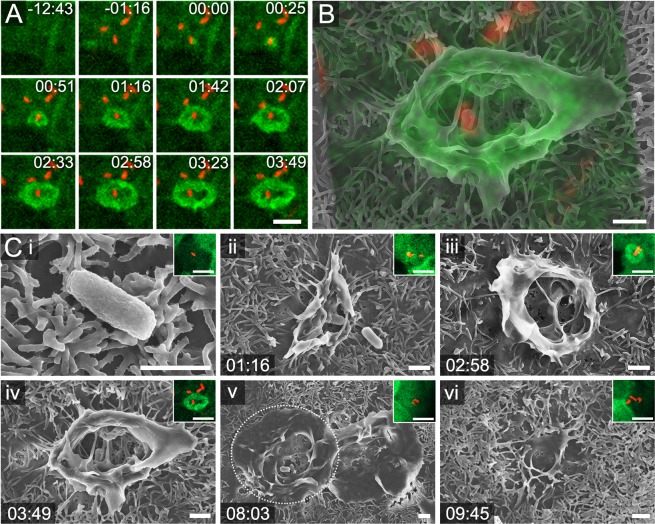
CLSEM for analyses of adhesion to, and invasion of polarized MDCK cells by *Salmonella enterica*. Polarized Lifeact-eGFP MDCK cells (green) were infected with STM WT (red). LCI was performed by SDCM and monolayers were fixed 17 min post infection (p.i.). (**A**) Time series showing the accumulation of F-actin into membrane ruffle in a frame interval of 25.43 s. The corresponding image sequence recorded over 17 min is shown in Movie 2. (**B**) Overlay of SDCM and SEM images for correlation of appropriate structures. (**C**) Representative morphological changes of the host cell membrane during adhesion and invasion of STM at individual ageing stages during point of fixation. (i) Early contact of STM to apical surface of MDCK. F-actin accumulation not yet visible. (ii) Triangular-shaped membrane ruffle (MR) already visible in SDCM. (iii) STM in SEM visible below a very thin carbon film. (iv) STM in the centre of MR. STM, which did not adhere tightly in SDCM, are no more visible in SEM. (v) MR retraction and reduced F-actin accumulation. Three STM were detected in SDCM, two STM were visible in the centre of MR in SEM modality, where later the Salmonella-containing vacuole (SCV) is formed. ROI is indicated by dotted circle. (vi) Complete internalization of STM into host cell in SDCM modality. Absence of MR and F-actin almost reorganized into brush border. Not all microvilli were fully recovered at this time point. Time stamp, min:sec. Scale bars, 1 µm.

Monolayer cells on gold mesh grids were infected by STM expressing mCherry and followed by fast LCI for periods of 15, 90, and 180 min post infection (p.i.). Although infection by bacterial pathogens imposes additional stress on host cells, monolayer integrity showed similar results as described above. We concluded that gold mesh grids are well suited for LCI of infection of epithelial monolayer cells. We visualized morphologic changes of apical brush border of Lifeact-eGFP MDCK cells during trigger invasion by STM (Fig. 5). Our workflow allowed precise correlation of fast LCI data with surface ultrastructure at a time point defined by chemical fixation. This enabled us to follow exactly the spatiotemporal dynamics of apical remodelling during infection of host cells. The visual time line demonstrates that translocation of SPI1 effector proteins through the SPI1-T3SS by STM into host cells led to distinct accumulation of F-actin at the apical entry side. Subsequently, STM was engulfed by membrane ruffles, accompanied by temporal disruption of the normal ordered structure of microvilli. Once STM resided in an intracellular vacuole, F-actin was reorganized and the original shape of brush border was restored. These cellular changes during invasion were completed in about 13 min after first bacterial contact. The data obtained can be used for further analyses defining the characteristics of adhesion to, and invasion of polarized epithelial cells. As an example, the increase/decrease of Lifeact-eGFP signal during STM invasion can be defined over time. By this, the final morphology visualized in the SEM can be correlated to the amount of F-actin accumulated by SPI1-T3SS effector proteins. Applying this to STM mutant strains, e.g. SPI1-T3SS effector mutants lacking one or more effector proteins, the specific role of each effector during invasion in polarized epithelial cells could be unravelled by morphometric analyses.

**Figure 6:**
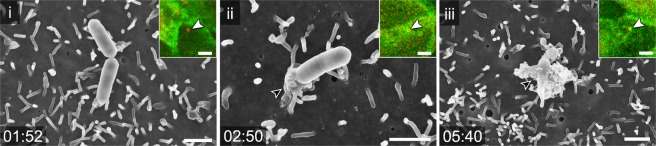
CLSEM to study adhesion to, and invasion of MDCK cells by *Listeria monocytogenes*. Shown is a timeline of morphometric changes of the host cell membrane during invasion of *L. monocytogenes* (red) in polarized Lifeact-eGFP MDCK cells (green). (i) *Listeria* adhered to microvilli on MDCK from time point 1:52 p.i., no increase of F-actin signal was visible. This matches lack of morphological changes in SEM. (ii) At 2:50 p.i., a small F-actin accumulation was visible (white arrowhead). In SEM, slight increase of membrane material appears at one side of bacterium (black arrowhead). (iii) At 5:40 p.i., fluorescence signals for F-actin and *Listeria* are both visible in SDCM (white arrowhead). The ultrastructure in SEM modality indicates that a small membrane protrusion had engulfed the bacterium (black arrowhead). Time stamp, min:sec. Scale bars, 1 µm (SEM), 5 µm (SDCM).

We applied our CLSEM workflow to other pathogens infecting enterocytes, such as *Listeria monocytogenes* and enteropathogenic *Escherichia coli* (EPEC). Due to interaction of *Listeria* invasin internalin A with epithelial surface protein E-cadherin, *Listeria* leads to internalization by the zipper mechanism and invasion occurs without recruitment of larger amounts of F-actin^6,7^. This invasion process of *Listeria* expressing mCherry into Lifeact-eGFP MDCK cells was barely detectable during LCI, since prominent F-actin accumulations were largely absent (Fig. 6). SEM micrographs deliver insights how the brush border was affected during invasion by *Listeria*. The visual time line shows that morphological changes of the apical membrane were more subtle, and invasion was faster compared to STM.

**Figure 7:**
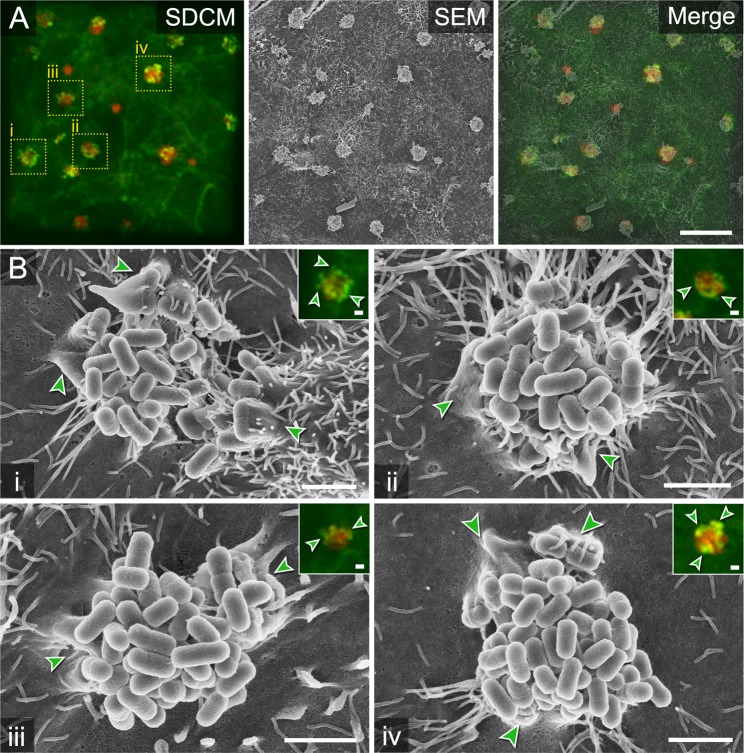
CLSEM of EPEC interaction with polarized epithelial cells. (**A**) Grid area with EPEC-infected (red) Lifeact-eGFP MDCK cells (green) imaged by SDCM, fixed 3 h p.i. with GA. Formation of EPEC clusters on the apical membrane with F-actin-enriched protrusions termed pedestals (light green spots), visible at sites of adhering bacteria. Processed samples were analysed by SEM using the InLens SE detector with EHT of 4 kV. Shape and distribution of EPEC clusters was used for defined correlation within relocated mesh holes. Selected ROI i-iv are indicated by yellow boxes. (**B**) SEM of clusters of EPEC at higher magnification of ROI i-iv. F-actin-enriched spots in SDCM modality correlate with pedestals visible in SEM modality (light green arrowheads). Scale bars, (**A**) 20 µm, (**B**) 2 µm.

During infection of epithelial cells, EPEC recruits F-actin into membrane extrusions called pedestals by translocating the effector protein Tir into the host cell membrane^9,10^. The T3SS required for Tir translocation and establishment of intimate attachment is expressed during co-culture of EPEC with host cells. Thus, LCI by SDCM of Lifeact-eGFP MDCK cells infected by EPEC expressing mCherry was performed 3 h p.i (Fig. 7). At this time point, clusters of EPEC adhering to the apical membrane were clearly visible. The clusters were associated with F-actin-rich foci. SEM micrographs of EPEC clusters revealed the presence of pedestal formation and tight membrane association with EPEC cells. Correlation of LCI and SEM modalities showed that F-actin-rich foci matched with pedestal formations of the host cell in contact with EPEC.

**Figure 8:**
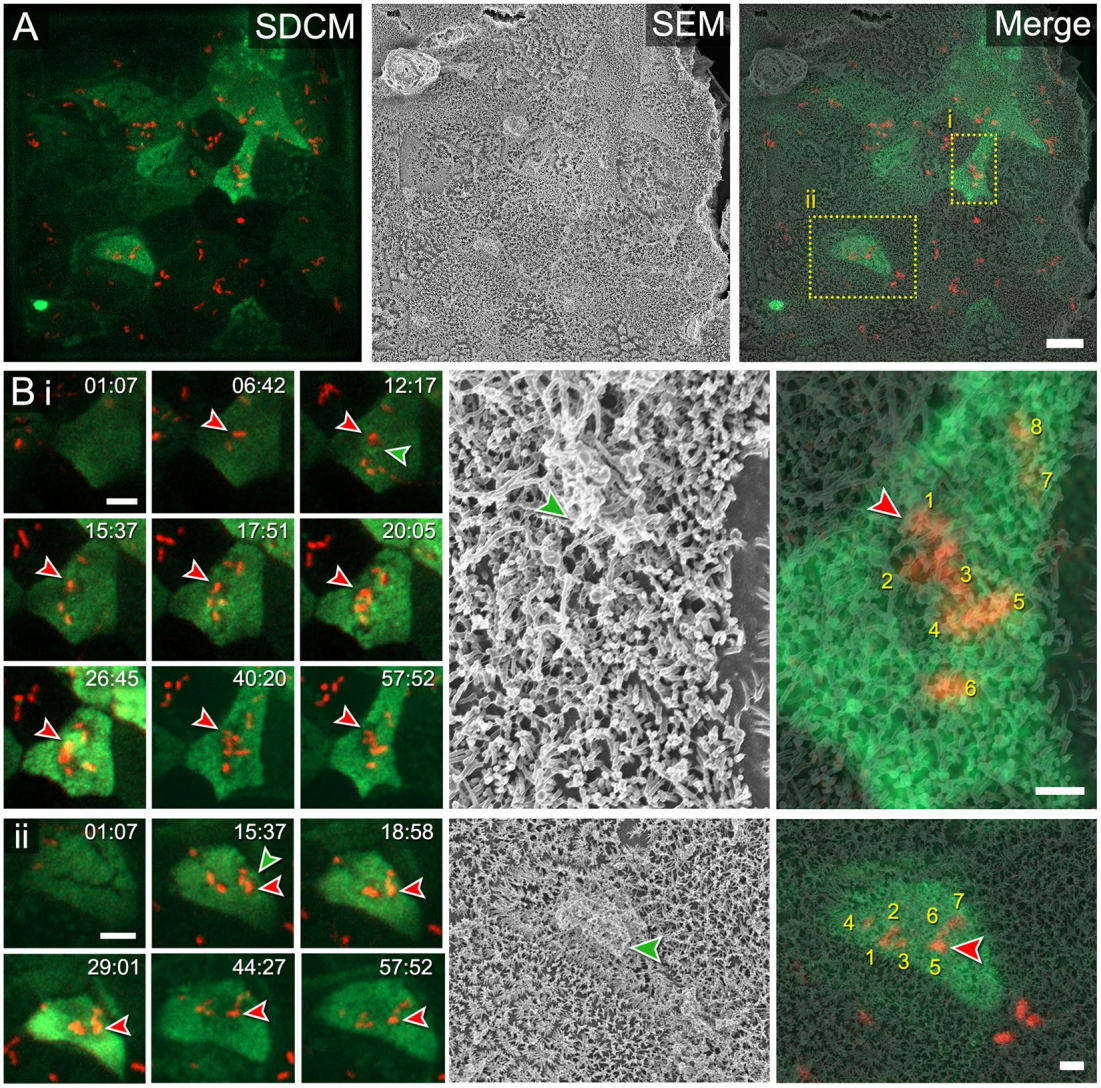
CLSEM for studies of brush border recovery after invasion of Caco-2 BBe1 cells by *Salmonella enterica*. (**A**) Mesh holes with STM-infected (red) Caco-2 BBe1 cells (green) imaged in SDCM modality, fixed 1 h p.i. by addition of GA. Single cells of monolayers show distinct recognizable shapes. Processed samples were analysed by SEM using the InLens SE detector with EHT of 1.8 kV. The final merging step was guided by the distinct shapes of Caco-2 BBe1 cells. The ROI (red dotted line) is shown in higher magnification in B i and ii. (**B**) Timeline of LCI shows successive STM invasion in Lifeact-eGFP Caco-2 BBe1 cells for 1 h. The fate of one STM bacterium was traced over time (red arrowhead in SDCM modality). Accumulation of F-actin into membrane ruffle (green arrowhead in SDCM) indicates effective invasion into host cell. Correlation to SEM modality reveals the total number of STM (numbers) that invaded into a host cell, and microvilli structure of the infected cell at time point of fixation. Multiple infection events are accompanied by changes in cell shape over time. (i) An arrow-shaped Caco-2 BBe1 cell was invaded by a total of 8 STM bacteria. At 1 h p.i., the host cell was devoid of F-actin accumulations in membrane ruffles (red arrowhead) and showed full recovery of the brush border (green arrowhead). (ii) A bean-shaped Caco-2 BBe1 cell was invaded by a total of 7 STM bacteria. This cell also lacks apical F-actin accumulation (red arrowhead). In SEM modality, a bulky array of membrane material is visible on the host cell surface at 1 h p.i. (green arrowhead), indicating an accumulation of apical membrane material that was not reorganized into normal brush border architecture. Time stamp, min:sec. Scale bars, (**A**) 10 µm, (**B**) SDCM 5 µm, SEM 1 µm.

While cellular dynamics like brush border rearrangement during the invasion process can be visualized by CLSEM in a convincing way, this also applies to surface analysis of later time points after bacterial invasion, as shown in Fig. 8. Here, we visualized the state of apical brush border of Lifeact-eGFP Caco-2 BBe1 at 90 min after infection by STM. Individual host cells were identified that harboured intracellular STM. These host cells showed complete restoration of the apical architecture and presence of microvilli (see Fig. 8Bi), as well as altered disturbed apical membrane organization and partial loss of microvilli (see Fig. 8Bii). Since the time points of entry of individual bacteria into a host cell of interest can be deduced from LCI, the apical morphologies can be precisely correlated to the infection process. Re-identifying distinct shapes of specific host cells emphasizes the robustness of this method for analysis of infection of polarized epithelial cell monolayers.

## Discussion

In this communication, we describe a workflow for correlative light and scanning electron microscopy (CLSEM) by applying gold mesh grids with C + F film as robust and convenient carrier for polarized epithelial cell monolayers. By using landmarks in the grid centres, cells grown in mesh holes can be chosen for fast LCI, and be relocated in SEM modalities to analyse the ultrastructure of cellular surfaces. Relocation of ROIs is based on the position of characteristic marks on the grids and correlation of modalities is performed by basic image analysis tools. Using this procedure, we combined data of fast live cell fluorescence microscopy with scanning electron micrographs to identify morphometric changes of apical brush border during bacterial infection.

Landmarks and mesh hole positions of the gold mesh grids are sufficient for combining standard fluorescence and scanning electron microscopes in a convenient and reliable manner, without the need of costly equipment or customized setups. However, this CLSEM method provides no automated correlation of images, relating to notes in electronic grid templates or overview images made during LCI to later identify specific mesh areas in SEM. For further enhancing correlative steps, pattern detecting plugins of open source software like FIJI/ImageJ should be tested. Metallic edges visible in fluorescence microscopy and SEM (by InLens and SESI) could be used as outlines to automatically superimpose images from different systems. This would further simplify the workflow, but was not tested so far.

Handling of mesh grids with C + F film has to be carried out with specific care, as the film is fragile and can be destroyed by tweezers or by washing steps too harsh. Outer mesh areas with relative long distance to the centre have a higher risk to be mechanically damaged during processing. Thus, it is advisable to choose mesh areas more centred, as they are saver located and easier to re-identify. To tackle this issue, gold mesh grids without C + F film could be used, which are irreversibly attached to dish bottom. Without a fragile film, defining the orientation of grids before cell seeding would be redundant, as monolayer cells would always grow in a pattern proposed in Fig. 1B. This could also lead to more stable focusing during fluorescence microscopy, as monolayer cells directly attach to dish bottom while growing in metal mesh, which is identifiable by LCI and SEM. We propose dishes with a glass bottom, which can be removed as cover slips, and can be transferred to standard SEM stab holders for analysis. By this, there would be no necessity of grid carrousel holder or STEM detector. Furthermore, one could try different gold grid types instead of mesh grids. For example, there is a large choice of EM finder grids with alphanumerical coordinates. This would spare the counting of mesh holes and make the whole grid easier accessible for CLSEM.

Another method to provide visible patterns directly on dish bottoms could be the use of micro-contact printing^19,20^. By this, cells are prevented to overgrow printed regions, leading to a specific pattern of cellular and non-cellular areas, which can be identified in both LCI and SEM. Performing preliminary tests, we observed that this may cause problems regarding attachment and differentiation behaviour of monolayer cells, especially the more sensitive Caco-2 BBe1 cells. MDCK cells, though, tend to overgrow printed borders when grid bars were spaced with mesh size of ≤200 µm. These problems could be addressed by widening mesh areas to provide more space for normal differentiation, and by expanding printed borders to prevent overgrowth. Future experiments should provide more data, if these suggestions raise bio-compatibility by using micro-contact printing.

Besides the pros and cons discussed so far, our method can be further expanded in versatility, for example by immuno-staining bacterial surface proteins^21^. After fixation, fluorescence antibody staining or an immuno-gold labelling can be applied to identify the fate of distinct surface structures. We already chose carbon coating for our grid sample processing, which is fundamental for immuno-gold staining. The SEM EsB (InLens energy selected backscatter) detector collects backscattered electrons of higher elements like gold, by which immuno-gold stained surface proteins can be analysed along conventional imaging with InLens or SESI detectors.

To further enhance the correlation accuracy, gold mesh grids can be combined with fiducial markers. Mohammadian, *et al*.^22^ applied silica coated gold nanoparticles to re-identify structures in both fluorescence microscopy and TEM images with an accuracy of 5 to 30 nm. This was not shown for SEM, though, but could provide the basis for high-throughput surface imaging with fiducial markers as additive landmarks to simplify following correlation steps manually or automatically. Similar approaches were already shown for CLEM in MDCK cells by 3D TEM tomography applying 15 nm protein A-coupled gold beads with a high accuracy below 100 nm^23^. Regarding SEM, even more sophisticated would be the application of mesh grid correlation for ultrastructural 3D imaging by focused ion beam SEM (FIB-SEM) or serial block face SEM (SBF-SEM). Samples embedded in epoxy resin can be cut in sections while imaging, ether by highly energetic gallium beam in FIB-SEM, or by an integrated diamond knife in SBF-SEM, leading to volume reconstruction. Beckwith, *et al*.^24^ proposed a workflow for correlative FIB-SEM of *Mycobacteria*-infected human macrophages. They applied thermo-deformed Aclar as pattern basis for orientation. Aclar is suitable for growth of monolayers cells and can be easily adjusted for experimental needs. By applying needle-engraved or carbon-coated grid patterns on Aclar^25^, FIB-SEM 3D tomography was performed on monolayer HUVEC cells. However, this approach was not tested for monolayer cells under fast LCI conditions. The thickness of Aclar could also affect focusing, but this should be tested with the available microscopic setups. Besides Aclar, gold mesh grids can be applied for 3D tomography imaging as well. By flat-embedding gold mesh grids in epoxy resin after ethanoic dehydration, the metal mesh can still be visualized with increased EHT beam, providing orientation for correlative FIB-SEM. For SBF-SEM, though, flat-embedding of EM grids is not advisable, as contact with metal could damage the sensitive cutting edge of the diamond knife. Instead, we would propose *en bloc* embedding with gelatine capsules as presented before^15^. When monolayer cells were grown on grids with C + F film facing downwards, EM grids can be removed and the grid pattern remains detectable in the resin. Another possibility for correlative FIB-SEM of cell monolayers could be the use of evaporated carbon patterns on glass bottom dishes, similar as for correlative high-pressure freezing and freeze substitution (HPF-FS) approaches^26^. By slightly adjusting our proposed workflow, further combinations with three-dimensional imaging and immuno-gold staining open up even more dimensions of future correlative data acquisition.

## Materials and Methods

### Culture of epithelial cells expressing Lifeact-eGFP

The generation of the Caco2 BBe1 Lifeact-eGFP cell line is described in Suppl. Material. The Lifeact-eGFP MDCK cell line was already described^4^.

For Lifeact-eGFP MDCK, 2 × 10^5^ cells per 35 mm glass bottom Petri dish (FluoroDish, WPI, No. FD35–100) were seeded in 1.5 ml MEM medium (Earle’s salts, 2.2 g × l^−1^ NaHCO_3_, without L‐glutamine (Biochrom, No. FG0325), non‐essential amino acids (NEAA, PAA, No. M11–003), 10% inactivated fetal calf serum (iFCS, Thermo Fisher Scientific, GibcoTM, No. 10270)) with additionally 100 U x ml^−1^ penicillin and 100 µg x ml^−1^ streptomycin (P/S, PAA), and cultured for five days. A confluent and polarized cell layer is formed at 2 × 10^6^ cells per dish. Medium was exchanged every second day.

For Lifeact-eGFP Caco-2 BBe1, 5 × 10^5^ cells per dish were seeded in 1.5 ml DMEM (4.5 g x ml^−1^ glucose, 3.7 g x ml^−1^ NaHCO_3_, with stable L‐glutamine, without Na-pyruvate (Biochrom, No. FG0435), 10 µg x ml^−1^ holo-Transferrin human (Sigma-Aldrich, No. T0665), 10% iFCS) with P/S. Lifeact-eGFP Caco-2 BBe1 cells were cultured for approx. two weeks to form a confluent and polarized cell layer with 1.8 × 10^6^ cells per dish. Medium was exchanged in intervals of two to three days. The day prior infection, cells were washed once in PBS, and the medium was exchanged to MEM or DMEM without P/S.

### Preparation of grids attached to dish bottoms

Cell culture material for CLSEM was prepared in a laminar flow cabinet, and 35 mm FluoroDish were used. A solution of filtrated 2% gelatine was preheated to 37 °C, and a 10 µl spot was put in centre of the glass bottom dish. A EM gold grid of 3.05 mm diameter with square holes of 200 µm^2^ mesh size and C + F film (Plano EM, No. S162A) was placed on top of the gelatine spot by forceps, then the excess gelatine was removed by pipette. Depending on the desired growth pattern of the cell monolayer, the C + F film was either facing upwards (see Fig. 1A) or downwards (see Fig. 1B). The almost invisible gelatine film was dried for 30 min at RT to adhere the grid to the glass bottom.

### Bacterial culture and host cell infection

*Salmonella enterica* serovar Typhimurium strain SL1344 (lab stock) harbouring pFPV-mCherry for constitutive expression of mCherry was used as wild-type strain for infection. The day prior infection, a single colony was inoculated for an overnight culture in LB broth with antibiotic selection by 50 µg x ml^−1^ carbenicillin at 37 °C in roller drum. On the day of infection, a subculture with ratio of 1:31 was grown at 37 °C in roller drum in LB broth with antibiotics for 2.5 h. OD_600_ was measured and bacteria were diluted to OD_600_ 0.2 in 1 ml PBS. Lifeact-eGFP MDCK and Lifeact-eGFP Caco-2 BBe1 cells were infected with MOI 5 to 10 directly on the microscope stage and after microscopy fixed at desired time points. For brush border recovery studies in Lifeact-eGFP C2BBe1, cells were infected for 30 min, washed thrice in fresh imaging medium, and imaged for another 60 min in imaging medium containing 100 µg x ml^−1^ Gentamicin before fixation.

*Listeria monocytogenes* strain EGD was kindly provided by Thilo Fuchs (Jena) and harboured pJEPAN4 for expression of HcRed^27^. A single colony was inoculated for an overnight culture in BHI medium with antibiotic selective erythromycin 5 µg x ml^−1^ at 37 °C in a roller drum. On the day of infection, overnight culture was diluted 1:3 and the subculture was grown for 1.5 h. OD_600_ was measured and subculture was diluted to OD_600_ 0.2 in 1 ml imaging medium without iFCS. Cells were infected with MOI of 100 directly on microscope stage.

Enteropathogenic *Escherichia coli* (EPEC) strain E2348/69 was kindly provided by Mathias Hornef (Aachen) and harboured pFPV-mCherry. A single colony was inoculated for over day culture in LB broth containing 50 µg x ml^−1^ carbenicillin at 37 °C with agitation in a roller drum. On the day prior infection, a static subculture with ratio of 1:51 was grown at 37 °C in DMEM (4.5 g x l^−1^ glucose, 3.7 g x l^−1^ NaHCO_3_, containing stable L‐glutamine and Na-pyruvate) without iFCS. On the day of infection, OD_600_ was measured and subculture was diluted to OD_600_ 0.2 in 1 ml PBS. Cells were infected with MOI of 30 for 3 h and fixed in 2.5% glutaraldehyde for 25 min at RT. Cells were washed thrice in 0.2 M HEPES and imaged.

### Live cell imaging by spinning disk confocal microscopy (SDCM)

Fast LCI was performed on a Cell Observer Z1 (Zeiss) fully motorized inverted microscope with Yokogawa Spinning Disc Unit CSU-X1a 5000 equipped with a custom-build acrylic glass incubation chamber. The incubation chamber of the system was heated at least 4 h prior infection in order to equilibrate to 37 °C. As objectives, Alpha Plan-Apochromat 63 × (NA 1.46, TIRF, oil immersion equipped with DIC slider EC PN 63 × 1.25 III, CA 63 × /1.2 W III), Plan-Apochromat 40 × (NA 1.4, DIC, oil immersion equipped with DIC slider CA 40 × /1.2 W, LD CA 40 × /1.1 W III), and Plan-Neofluar 10 × (NA. 0.3, DIC I, Ph 1, air) were used. The system was equipped with 120 W metal halide fluorescence lamp HXP 120 C from Zeiss with internal electronic shutter, and with Zeiss computer-controlled multi-colour laser module with AOTF combiner (405 nm diode laser, max. power 50 mW, 488 nm optically pumped semiconductor laser, max. power 100 mW, 561 nm diode laser, max. power 40 mW, 635 nm diode laser, max. power 30 mW). Two Photometrics Evolve EMCCD cameras were used in DualCam setup on the spinning disc unit. The light paths for left and top cameras contained filters for eGFP (Zeiss Filter set 38 HE) and Cy3 (Zeiss Filter set 43 HE), respectively. An area of 512 × 512 pixels with 212 nm per pixel (63x objective) was imaged with laser power of 5% to 10% for 488, and 7% to 12% for 561, both channels with 25 ms exposure time. Acquisition of data was done in Zeiss ZEN 2012 (blue edition), data were stored in the OMERO 5.3.5 database system, and images were processed by FIJI/ImageJ 1.52 h and Adobe Photoshop CS6.

### Preparations before infection of cells on gold mesh grids on microscope stage

Medium in FluoroDish was exchanged by 1 ml imaging medium (MEM with Earle’s salts without NaHCO_3_, L-glutamine, phenol red (Biochrom, No. F0385), with additionally added 30 mM HEPES). For infection with *Salmonella*, 10% iFCS was added to imaging medium, whereas for *Listeria*, imaging medium without iFCS was used. The FluoroDish with cells was transferred to a pre-heated microscope stage and the lid was removed before defining grid areas to reduce the risk of losing the image area during fixation by touching. To avoid delays before LCI, grid areas with ROIs were defined at least 1 h prior infection. With a 63x oil objective, the centre mesh area with a single small notch was located for orientation during imaging. In case of multi-position imaging, the centre mesh with notch was used as landmark, and other locations were marked on grid template in regard to the landmark. Acquisition settings (z-stack, exposure time) for fast imaging were defined, depending on dynamics of structure of interest. As for *Salmonella*-induced membrane ruffling, the time gap from frame to frame was ≤30 s. Infection was done directly on the microscope stage for infection times ranging from 15 min up to several hours.

### Chemical fixation, dehydration and chemical drying of infected cells

Double-concentrated fixative, i.e. 4% glutaraldehyde (GA) in 0.4 M HEPES buffer was freshly prepared and pre-heated to 37 C. An equal volume of fixative was carefully added to cells in imaging medium while imaging was continued. Care was taken to avoid detaching the grid from the bottom of the FluoroDish. Fixation by 2% GA led to dramatically increased background fluorescence. The fixation process was recorded for another 10 min on stage until fully fixed morphologies were reached. The FluoroDish was transferred to a fume hood, and fixative mixture was replaced by 1 ml fresh 1-fold GA fixative for 15 min at RT. Then, cells were rinsed thrice with 1.5 ml 0.2 M HEPES buffer. Dehydration was done in graded ethanol series at RT by 10%, 30%, 50% ethanol once for 10 min each, and 70%, 90%, 100% ethanol twice for 10 min each. For chemical drying, hexamethyldisilazane (HMDS, Sigma-Aldrich, No. 440191) was used with pure ethanol in freshly prepared ratios of 1:3, 1:1, 3:1 once for 15 min at RT, and 100% HMDS once for 20 min at RT. After 100% HMDS, liquid was discarded completely. The FluoroDish with EM grid was air-dried for at least 30 min at RT in a fume hood until all liquid has evaporated. Samples were stored in a desiccator with silica gel orange beads.

### Immunofluorescence imaging of tight junction formation

Epithelial monolayer cells grown on mesh grids in dish bottoms were fixed in methanol at −20 °C overnight, then washed thrice in PBS at RT. Antibodies were diluted in blocking solution (2% goat serum, 2% bovine serum albumin in PBS) and applied to staining at RT. Samples were incubated with primary antibody rabbit α ZO-1 (1:200, ThermoFisher Scientific) for 2 h and washed thrice in PBS. Secondary antibody goat α rabbit Alexa594 (1:500, Dianova) was applied for 1 h and washing steps were repeated. Samples were mounted in Fluoroshield and sealed with Entellan (both Merck). Imaging was done by SDCM with ZEN 2.6 software. Additional image processing was done in FIJI/ImageJ v.1.52p (National Institutes of Health USA) and Photoshop CS6 (Adobe).

### Imaging by scanning electron microscopy for correlative workflows

Grid samples were further prepared for high-resolution field-emission scanning electron microscopy (FESEM) (Auriga CrossBeam, Zeiss) with integrated high-resolution focused ion beam (FIB) milling with SmartSEM 5.07 software. First, the grid sample was carbon-coated using a Bal-Tec MED 020 modular high-vacuum coating system. Using forceps, grids were transferred to a Zeiss carrousel sample holder for EM grids. The carrousel sample holder was placed onto the SEM motorized stage, and the vacuum chamber was evacuated. A Schottky field emission Gemini electron column (EHT, electron high tension) was used between 1.8 kV to 4 kV for imaging, and up to 15 kV to visualize the metal mesh. As detectors, InLens SE (secondary electrons), SESI (secondary electrons secondary ions), and STEM (scanning and transmission electron microscopy) detectors were used. When an area with orientation marks was located, the detector was switched to InLens SE with EHT of 1.8 kV to 4 kV. To re-identify areas besides the centre area, meshed areas were localized according to the grid template, where notes haven been done during fluorescence LCI. For high-resolution imaging of cell areas larger than 50 µm², ATLAS 4.0 software (Zeiss) was used to automatically merge single micrographs to a complete tiled image. Images of SDCM and SEM modalities were correlated manually in Adobe Photoshop CS6.

## Supplementary information

Suppl. Materials

Movie 1

Movie 2
