# Supplementary material for: Correlative light and scanning electron microscopy (CLSEM) for analysis of bacterial infection of polarized epithelial cells
Source: Sci Rep. 2019 Nov 19;9:17079. doi: 10.1038/s41598-019-53085-6 (PMC6863815; doi:10.1038/s41598-019-53085-6)
Supplement: Supplementary file 1 — Suppl. Materials [file 41598_2019_53085_MOESM1_ESM.pdf]

Correlative light and scanning electron microscopy (CLSEM) for analysis of bacterial infection of polarized epithelial cells

Carina Kommnick[1], Andrea Lepper[1], and Michael Hensel[1,2]

[1] Abt. Mikrobiologie, [2] CellNanOs – Center for Cellular Nanoanalytics Osnabrück, Fachbereich Biologie/Chemie, Universität Osnabrück, Osnabrück, Germany

Suppl. Materials and Methods

Lentivirus production for Lifeact-eGFP transduction

Production of lentiviruses for Lifeact-eGFP transduction was done according to protocols in 'The RNAi Consortium (TRC) Laboratory Protocols. Lentivirus production of shRNA, CRISPR, or ORF-pLX clones in 10 cm dishes or 6 well plates' (Version: 03. June 2015) with adjustments. 3.8 x 10^6 HEK 932T cells were seeded per 10 cm dish in DMEM without P/S + 10 % iFCS (inactivated Fetal Bovine Serum, Thermo Fisher Scientific, GibcoTM, No. 10270). psPAX2 (lentiviral packaging plasmid), pMD2.G (lentiviral envelope plasmid), and pLX304 (expression of transgene) were obtained from Addgene. Transfection was done by FuGene reaction mix with 270 µl FuGene, and 450 µl OptiMEM Pro for 5 x 10 cm dishes with 144 µl per dish, incubating for 5 min at RT. For production of lentiviral particles, 9 µg packaging plasmid psPAX2, 0.9 µg envelope plasmid pMD2.G, and 9 µg pLX304 Lifeact-eGFP were incubated with FuGene mix for 30 min at RT, then transferred onto HEK 293T cells, and incubated for 20-24 h at 37 °C, 5 % CO_2 in a humidified incubator. On the next day, the medium was exchanged to DMEM containing 100 U x ml^{-1} penicillin and 100 µg x ml^{-1} streptomycin (P/S, PAA), and 30 % iFCS, and cells were incubated for another

20-24 h. After this, lentiviruses in the supernatant were collected, filtered in 0.22 µm filters, aliquoted in 2 ml reaction tubes, and stored at -80 °C. Fresh DMEM containing P/S and 30 % iFCS was transferred onto the cells for another day, then lentivirus particles were collected again as described. After the second lentivirus harvest, HEK 932T cells were discarded.

Generation of Lifeact-eGFP Caco-2 BBe1 cell line

Caco-2 BBe1 cells were seeded in 24 well plates with 2 x 10^5 cells per well in 500 µl DMEM w/o P/S containing 10 % iFCS, 0.01 mg x ml^{-1} holo-Transferrin (HT), and incubated 20-24 h at 37 °C, 5 % CO_2 in a humidified incubator. On the following day, DMEM medium was removed, and 350 µl of fresh DMEM was added per well. 5-15 µl of lentiviral particle suspension were transferred into wells, and mixed by gently pipetting. The lentiviral infection was incubated 14-18 h at 37 °C, 5 % CO_2 in a humidified incubator. To increase transduction efficiency, 8 µg x $µl^{-1}$ Polybrene was added to the cells. On the next day, medium with lentiviral particles was removed, and 500 µl fresh medium was added. Cells were grown for another 2 days, when medium was removed again, and fresh medium with concentration of 10 µg x $µl^{-1}$ Blasticidin was added for antibiotic selection of transduced marker in the target cell genome. To define an appropriate concentration for Blasticidin in Caco-2 BBe1, an antibiotic kill curve was performed before. Medium with Blasticidin was replaced every 3 days, until expression of Lifeact-eGFP was visible in some positively transduced cells via fluorescence microscopy. Cells were treated with Biotase (Biochrom, No. L2193) to detach them from wells, and transferred into 6 well plates in 2 ml medium with Blasticidin. When grown to 80 % confluency, cells were treated with Biotase, and a serial cell dilution in 96 well plates with 0.7 cells per well in 180 µl medium was performed. Therefore, medium mix with 70 % fresh DMEM containing P/S, 10 % HT, and 30 % spent supernatant of Caco-2 BBe1 cells was used. Wells of 96 well plates were checked for single cells the next day. Single cells were grown over several weeks to monolayer of 80-90 % confluence, where Lifeact-eGFP expression reaches a level visible by fluorescence microscopy. Transduced cells were detached with Biotase and

transferred in 24 well plate (500 µl), and checked for homogenous and stable expression of Lifeact-eGFP. Populations of successfully transduced and stably expressing Lifeact-eGFP cell lines were expanded in 6 well plates (2 ml), and in small flasks (5 ml in 25 cm^2), when reaching 80-90 % confluency. The Lifeact-eGFP MDCK cell line was already described by Lorkowski, et al. [4].

Suppl. Figures

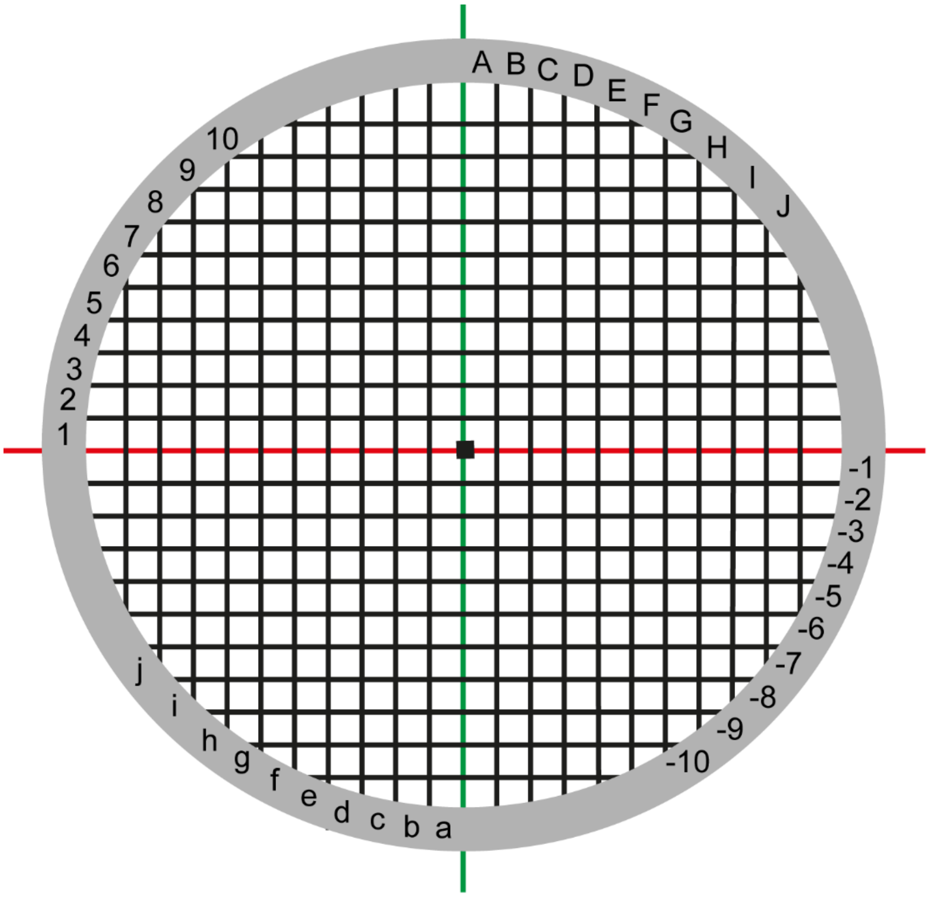

Fig. S 1. Template for coordinates of grid holes in mesh grid 200.

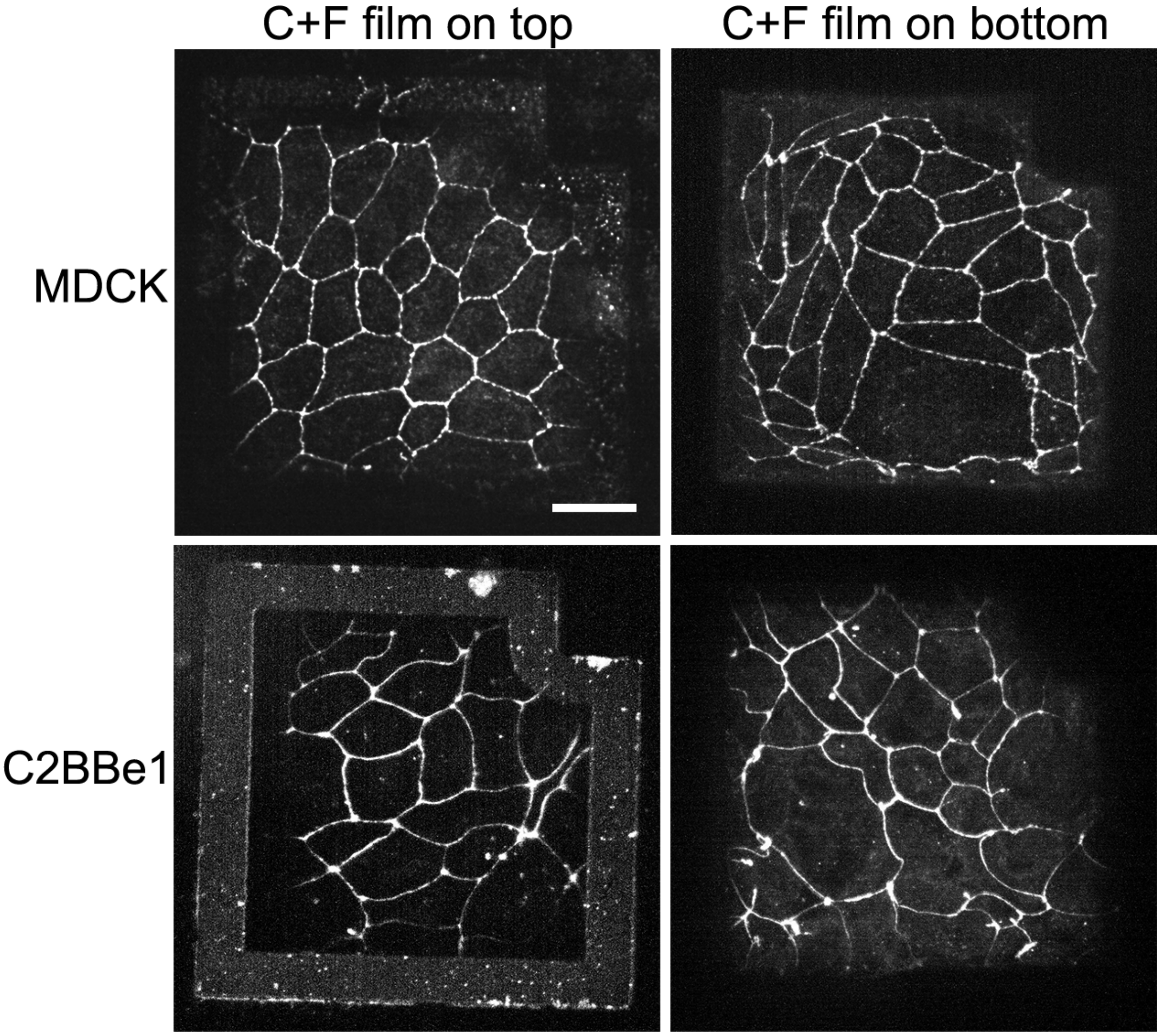

Fig. S2. Analyses of polarized epithelial monolayer and tight junction formation on gold mesh grids by immunofluorescence labelling of zonula occludens-1 protein (ZO-1). Formation of polarized epithelial monolayer was verified by ZO-1 staining and Alexa594 labelling in MDCK cells (5 days of growth) and C2BBe1 cells (19 days of growth). Both orientations of C+F film were tested, showing no differences in monolayer formation. Scale bar, 20 µm.

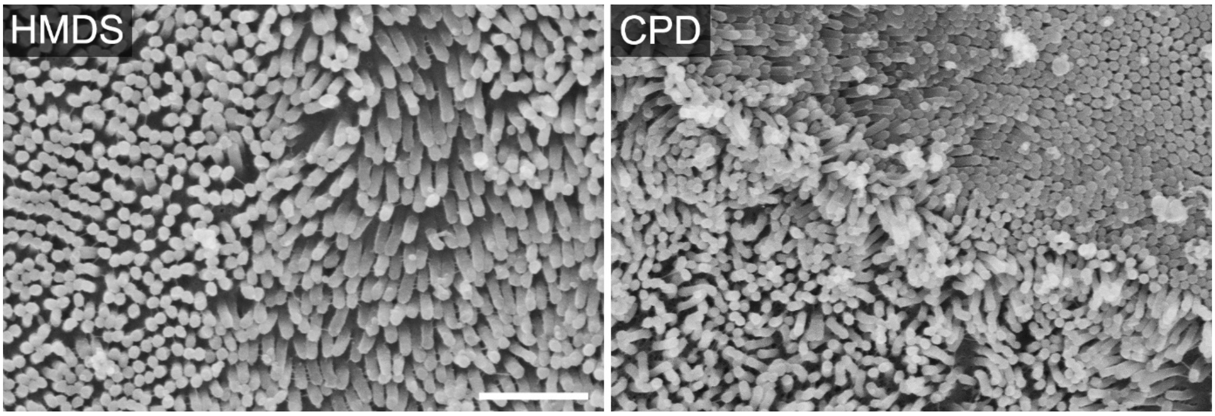

Fig. S3. Comparison of brush border preservation after chemical drying with HMDS or critical point drying. C2BBe1 cells were grown for 19 days on glass coverslips, fixed by GA 2.5 % in PBS for 20 min, and dehydrated in a graded ethanol series as described in the main text. The final drying step was performed either by chemical drying using HMDS, or critical point drying (CPD) was applied to samples using a Leica EM CPD300 instrument according to manufacturers' instructions. Samples were carbon-sputtered and analysed by SEM using the Auriga FESEM system (Zeiss). Scale bar, 1 µm.

Suppl. Movie Captions

Movie 1. Live cell imaging of infection. MDCK cells expressing Lifeact-eGFP (green) were infected with STM WT expressing mCherry (red). MDCK cells were grown of gold mesh grid and infection was performed on the stage of the SDCM. The movie corresponds to the still image shown in **Fig. 2**C and represents a time lapse sequence of 17 min. Time stamp, min:sec.

Movie 2. Live cell imaging of infection. Infection of MDCK cells expressing Lifeact-eGFP (green) with STM WT expressing mCherry (red) was performed as for **Movie 1**. Details of STM interaction with one host cell are shown. The movie corresponds to the still image shown in **Fig. 5**A and represents a time lapse sequence of 17 min. Time stamp, min:sec.
